# Does randomised evidence alter clinical practise? The react qualitative study

**DOI:** 10.1186/s12913-024-11305-4

**Published:** 2024-07-29

**Authors:** Samuel Lawday, Karen Mattick, Rob Bethune

**Affiliations:** 1 HeSRU, Royal Devon & Exeter Hospital, Exeter, UK; 2 Department of Health and Community Sciences, Faculty of Health and Life Sciences, University of Exeter, Exeter, UK; 3 Centre for Surgical Research, Population Health Science, Bristol Medical School, University of Bristol, Bristol, UK; 4 North Bristol NHS Trust, Bristol, UK

**Keywords:** Surgery, Evidence, Trials, Implementation

## Abstract

**Background:**

In 2015, the results of the ‘Small bites versus large bites for closure of abdominal midline incisions (STITCH) Trial’ were published in The Lancet. This demonstrated the superiority of small bite laparotomy closure over mass closure for the reduction of incisional hernias; despite this most surgeons have not changed their practice. Previous research has shown the time taken for the implementation of evidenced based practise within medicine takes an average of 17 years. This study aims to understand the reasons why surgeons have and have not changed their practice with regards to closure of midline laparotomy.

**Methods:**

Semi-structured interviews were completed with surgical consultants and registrars at a single institution in South West England. The interview topic guide was informed by a review of the published literature, which identified barriers to adopting evidence into surgical practice. Interview transcripts underwent thematic analysis with themes identified following discussions within the research team, exploring views on published data and clinical practise.

**Results:**

Nine interviews with general surgical and urological consultants as well as registrars in training were performed. Three themes were identified; ‘Trusting the Evidence & Critical Appraisal’, ‘Surgical Attitude to Risk’ and ‘Adopting Evidence in Practise’, that reflected barriers to the introduction of evidenced based practise to clinical work.

**Conclusion:**

Identification of the themes highlights possible areas for intervention to decrease the adoption time for evidence, for example from randomised controlled trials. The continued updating of clinical practise allows clinicians to provide best evidenced based care for patients and improve their outcomes.

**Supplementary Information:**

The online version contains supplementary material available at 10.1186/s12913-024-11305-4.

## Background

Surgical practise has mostly shifted from being based upon ‘comic opera’, surgical dogma and case series, to well conducted cohort studies and randomised controlled trials (RCTs) [1]. Over this time, surgeons have produced data to allow a more evidence-based approach to clinical practise. This evidence has highlighted alternative management strategies for disease and different surgical techniques, which can improve patient outcomes such as post-operative morbidity, mortality, oncological and quality of life.

There is an average 17 year delay for the implementation of evidenced based practise, delaying potential clinical benefits to patients [2, 3]. This time from publication of RCTs to the widespread adoption is surprisingly long and estimates also show that only half of EBM is adopted into general clinical practice [4, 5]. Issues with the adoption of EBM can be due to the research itself (e.g. inappropriate research questions, inappropriate methods, inaccessibility of a paper, biased findings or unusable reporting [6]) or due to other clinical factors (different patients population or variations in current clinical pathways).

In 2015, the results of the ‘Small bites versus large bites for closure of abdominal midline incisions (STITCH) Trial’ were published in The Lancet [7]. This was a multi-centre, double-blinded RCT investigating closure method following mid-line incisions for elective surgery. They compared small bite (SB) closure, the same technique used in a previous single centre RCT, against standard practice. Five hundred and forty five patients were included in the final analysis and the study demonstrated that, with SB closure, patients had a reduced incidence rate of incisional hernia at 1 year (*p* = 0.03). Since the publication of the STITCH trial, meta-analysis supported the finding of reduced incisional hernia rate with the use of SB closure [8]. The European Hernia Society (EHS) guidelines in 2015 and 2022 recommended SB technique for fascial closure [9, 10]. A recent BJS editorial also supported the use of SB closure and highlighted its importance of closure as part of clinical care [11]. Despite this, few surgeons seem to have changed their clinical practise [7, 12, 13].

This study set out to understand the reasons why surgeons have and have not changed their practice with regards to closure of midline laparotomy; with the expectations that many of these findings may be transferable to other areas of surgical practice.

## Methods

### Aim

This study aimed to explore the perceptions of surgeons on the underlying reason behind the adoption or rejection of small bite closure into routine clinical practise.

### Study design

This study explored clinician perceptions using qualitative semi-structured interviews. A scoping review of published work was completed to inform the interview topic guide. This identified knowledge of the evidence, belief in the evidence, resources and patient factors as possible barriers to implementation of evidenced based surgical practise. The topic guide was trialled and subsequently modified within the research group prior to use (Appendix 1). Closure technique was discussed for open surgery using midline laparotomies but also for midline extraction sites following laparoscopic surgery.

### Participants and setting

The research setting was a hospital in the Southwest of England. The hospital was a large district general hospital with tertiary colorectal services and on-site urology.

Inclusion criteria of the study were:

- Surgical consultant or registrars.
- Surgical specialities including but not limited to general, vascular, gynaecological and urological surgeons.
- Perform midline elective laparotomy closure as part of routine clinical duties.

Purposive sampling of surgeons was completed. Surgeons were contacted by e-mail following identification through the on-call rota with a participant information leaflet. Face to face interviews were conducted on site at a mutually convenient time following the acquisition of written consent.

### Data collection

Interviews took place between the June and July 2019 in a private room and were audio-recorded. These underwent professional transcription, after which the audio recording was deleted. Basic demographic details were recorded at the start of the interview prior to discussion about the use of small bite closure and evidence use in clinical practise. Recognising that ‘thematic saturation’ is a contested concept, we undertook sufficient interviews to answer the research question in the single setting, and to start to explore transferability to other contexts, whilst recognising and accommodating the significant time pressures on the participants [14].

### Data analysis

Transcripts underwent thematic analysis using NVIVO software [15]. An inductive approach was taken with no specific theory in mind; the scoping review had informed the design of the topic guide, however this was not taken into account during the analysis. Codes were produced from the analysis and grouped into themes; this underwent multiple iterative and sequential changes following further analysis of the original codes and discussions within the authorship group. The manuscript was written in accordance with Standards for reporting qualitative research (SRQR) reporting guidelines [16].

### Team reflexivity

The interviewer was a junior surgical trainee and was well known to those who were interviewed having worked in the department for 2 years. This may have affected the information that surgeon participants were willing to give during the interview. The primary author prior to analysis undertook structured external qualitative training to ensure validity and quality of the analysis. The research team consisted of a surgical trainee, a surgical consultant with qualitative experience and an experienced qualitative researcher.

The surgical consultant had already changed their practice to SB closure before the STITCH trial was published and so the results fitted into to their prior beliefs. This attitude may have reflected in the attitude of the surgical team and may have affected how some surgeons who didn’t use SB closure viewed the research project.

### Ethical approval

for this project was sought and obtained by the ethics committee of the NHS Health Research Authority (IRAS 255,295) & the University of Exeter (RG/CB/19/4/210). All participants gave their written consent.

## Results

### Interviews

Nine interviews were completed, with 11 different surgeons having been approached. One surgeon not interviewed did not meet inclusion criteria and the other a suitable time for the interview could not be made. Eight surgeons were colorectal surgeons and one was a Urologist. No upper gastro-intestinal surgeons at the site met the inclusion criteria. This represented greater than 50% of eligible general surgical consultants. Three surgeons were registrars who had completed a minimum of 6 years post graduate surgical training and one was a fellow who had completed their general surgical training abroad but was completing a year of higher level training. The remaining five were consultants with a variety of experience, with completion of surgical training varying from 2000 to 2016. One interviewee had an academic career but the other participants had no or minimal active academic involvement.

All participants completed the interview. Average duration of the interviews was 22:49 min (range 14:20 to 36.37). Demographics can be found in Table 1. The codes from thematic analysis were reviewed at successive time-points. Following the 9th interview, the research team deemed further interviews within this cohort at a single site unlikely to identify any further themes to aid answering the research question. Further interviews were therefore stopped as theoretical sufficiency had been determined [14].

**Table 1:** Participant demographics

Demographics	*N*
Date of graduation	1988–2008
Year of completion of surgical training (if applicable)	2000–2016
**Stage of career**	
Registrar	3
Fellow	1
Consultant	5
**Speciality**	
General surgery (upper gastrointestinal surgery)	0
General surgery (colorectal surgery)	8
Urology	1
**Research involvement**	
University professor	1
Postgraduate degree (completed or current)	6
On-going research	8
**Use of small bite closure**	
Use routinely	3
Use occasionally	3
Do not use	3

### Themes

**Fig. 1:**
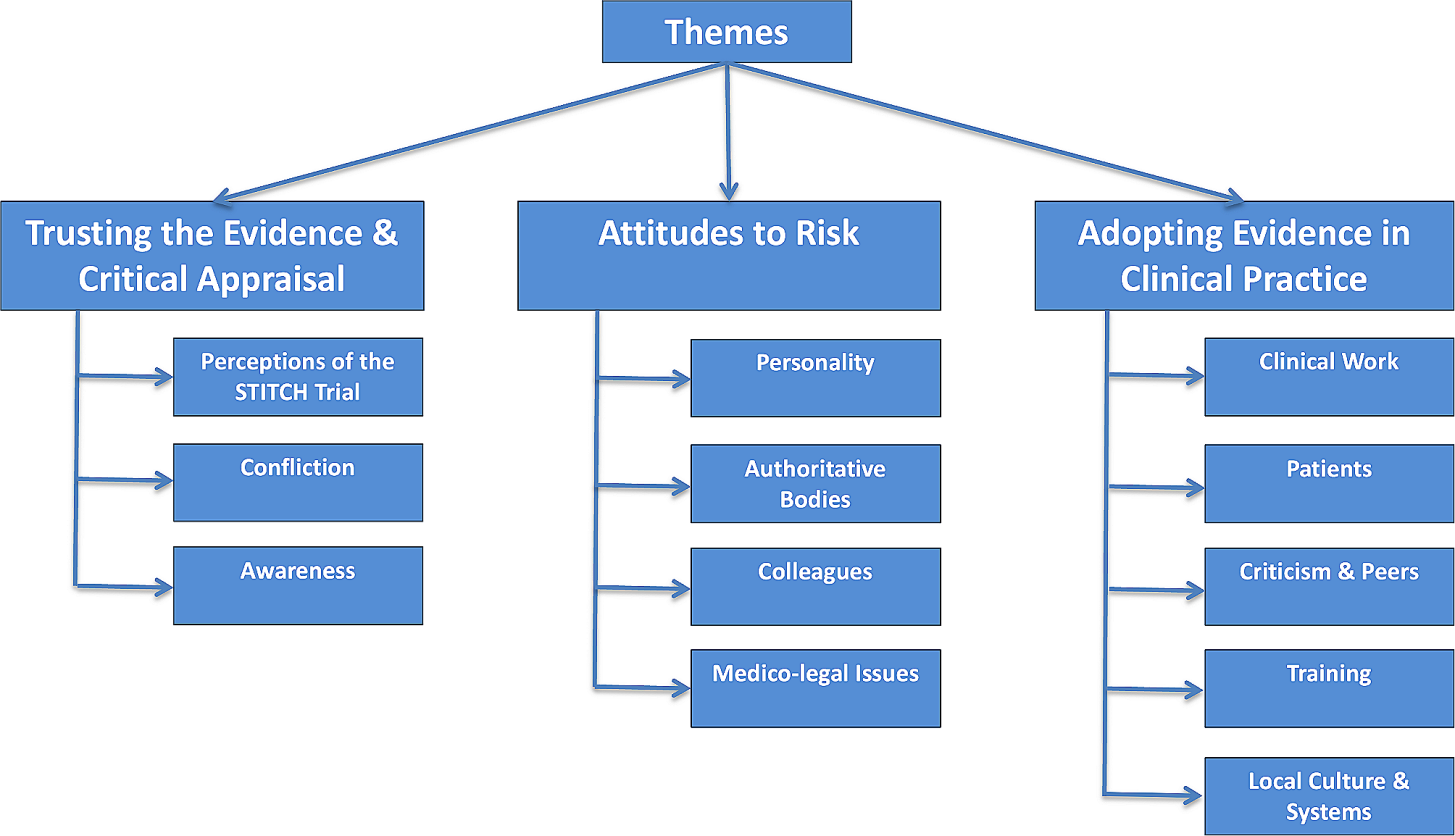
Themes The themes and grouped codes identified following thematic analysis

Three themes were identified following the thematic analysis of the interview transcripts. These were ‘Trusting the Evidence & Critical Appraisal’, ‘Surgical Attitude to Risk’ and ‘Adopting Evidence in Practise’ (Fig. 1). Quotes are reported with a unique identifier and surgical grade (C = consultant and R = registrar).

### Theme 1: ‘Trusting the evidence & critical appraisal’

This theme covered different codes identified within the literature but these focused on the STITCH trial itself. Part of the issue of adoption into clinical practise was a lack of awareness of the data.

*“I knew about the STITCH trial before but hadn’t really read it” (Interviewee 783*,*R)*.

Those who were aware of it had different perceptions of the trial data. Some surgeons thought this was a high quality trial and justified the use of SB closure as part of their clinical practise. However, others thought the wrong question had been asked and were therefore less willing to take on this new technique.

*“What those original trials looked at was comparing a technique which I don’t use*,* with a technique which I don’t use.” (Interviewee 412*,* C)*.

*“the view amongst us is that it is not the small bite itself*,* but what makes a difference is the definition of the anterior sheath” (Interviewee 463*,* R)*.

The conflict within the literature and that consultants did not consistently use the same technique was a point of frustration for one registrar. They found this conflict difficult to then decide what should be done in their own practise.

*“it’s really frustrating reading all the different papers and then speaking to all these different consultants who all know what they’re talking about .…… even though it’s completely at conflict with what someone else believes” (Interviewee 186*,* R)*.

### Theme 2: attitude to risk

This theme reflected how different surgeons responded to the risk that is inherent in their clinical practise and therefore influenced the uptake of SB closure.

The varying characteristics of surgeons were reported to be a key influencer in the uptake of the new technique and this was recognised by the surgeons interviewed. There was also a recognition that surgeons who continued to modify their practise found this easier; surgeons who did not make regular changes to how they practised clinically found making adaptions much harder.

*“I mean………. it comes down to personalities*,* as well …… There are definitely some people who are more likely to pioneer new things” Interviewee 543*,* R)*

*“I think I’m more cynical” (Interviewee 412*,* C)*.

*“I’m probably a bit of a luddite in terms of new things” (Interviewee 911*,* C)*.

*“I think once you feel that you are doing something the optimum way…. I think it’s more difficult to change” (Interviewee 704*,* R)*.

The role of authoritative bodies in managing the risk for the individual surgeons was also raised by those interviewed. Some surgeons felt that if a wider or expert group supported a new technique, they would be more likely to use it. This reflects the shift of risk for the individual surgeon to an authoritative body, if it supported their practise. It wasn’t just authoritative bodies that reflected a shift of risk. Surgical colleagues, both locally and nationally, utilising a new technique meant other surgeons left safer and more supported using such a technique:

*“if there had been a diktat from the college or somewhere to say*,* thou most close laparotomies using a small bite closure*,* then you would have no choice” (Interviewee 911*,* R)*.

*“I say*,* for me*,* I’m probably being a bit of a chicken*,* if I was on my own*,* the only person doing a particular closure*,* let’s say*,* then that would worry me” (Interviewee 911*,* C)*.

*“in consideration with the department I’m working in. I wouldn’t do anything necessarily out on my own” (Interviewee 783*,* R)*.

The perceived risk associated with adopting a new technique was also linked to medico-legal issues by many of the interviewees:

*“If you’re going to adopt a new technique you have to be able to justify it to yourself because if you can’t justify it to yourself*,* you certainly won’t be able to justify it to the patient or to the patient’s lawyer if something goes wrong. That’s key really” (Interviewee 412*,* C)*.

### Theme 3: adopting evidence in clinical practice

The third and final theme was relevant to the barriers within clinical environment that meant surgeons were not able to alter their practise. Surgeons discussed the use of anecdotal evidence of the patients and results they saw on a day to day basis and how that had a substantial impact on the techniques they use.

*“it is evidence-based as well as anecdotal evidence based on your own practice” (Interviewee 463*,* R)*.

*“it became more apparent that the way that seemed to be working best was to just take the anterior rectus sheath” (Interviewee 412*,* C)*.

*“we weigh our own personal experience against what we*,* I guess*,* know to be true*,* from all what we believe to be- whether or not we believe it to be true from the research” (Interviewee 543*,* R)*.

*“If I start doing small bite closure and then I get a wound dehiscence*,* I’ll be mortified.” (Interviewee 412*,* C)*.

There was also some concern that novel surgical techniques were not acceptable for patients. Surgical apprehension also centred on the new technique not being appropriate for all patients; the original trial had strict inclusion and exclusion criteria and therefore the evidence is not there for a large proportion of patients undergoing midline laparotomy.

*“if you’ve maybe told a patient that you’ve tried something new and then they’ve had a problem with it. I think it feels very different” (Interviewee 543*,* R)*.

*“if you go to a patient and say*,* everybody does it this way*,* they’re automatically happier. I think selling evidence of patients if very difficult” (Interviewee 911*,* C)*.

There was also unease regarding the training surgeons undergo. In part, this was clinical. Surgeons felt they had not been taught about the new SB technique and therefore did not feel comfortable utilising it in clinical practise:

*“I mean*,* I’m aware of the backdrop and the evidence-base to this project*,* but that doesn’t really come into my mind*,* because I don’t feel that anyone has*,* like*,* sat me down and taught me about it” (Interviewee 186*,* R)*.

*“So*,* I think that’s a technical aspect of it*,* but unless it’s specifically taught to you*,* or you specifically think about it” (Interviewee 543*,* R)*.

Surgeons also discussed that there was minimal education during surgical training pertaining to the translation of evidence into clinical practise. The use of journal clubs and exams mainly focused on critical appraisal of a single journal article and not the impact this has on clinical practise:

*“In terms of how you then translate that to everyday practice*,* probably not an awful lot” (Interviewee 911*,* C)*.

*“translational stuff from research to clinical practice*,* I’ve not had specific training about” (Interviewee 186*,* R)*.

The local culture that surgeons worked in was also highlighted as a potential barrier to the implementation of the new technique:

*“So*,* again*,* that kind of almost comes back to what you were saying earlier about culture*,* and if other people are changing*,* it makes it easier for you to change” (Interviewee 543*,* R)*.

*“I think it’s probably definitely a changing culture. I think some of the more senior consultants; it would take a lot to get them to change their practice. So*,* yes*,* I think things are changing” (Interviewee 704*,* R)*.

*“there were some dinosaurs there …… the old guards would not change their ways*,* regardless” (Interviewee 783*,* R)*.

## Discussion

This study set out to understand the reasons why surgeons have and have not changed their practice with regards to closure of midline laparotomy, as a model for the implementation of evidenced based practise into routine clinical practise. Semi-structured qualitative interviews underwent thematic analysis led to the identification of three barriers to the adoption of the new technique. ‘Trusting the Evidence and Critical Appraisal’ focused mainly on the individual interpretation of the results of STITCH trial. Issues with outcomes, length of follow up and the comparator meant take up of the new technique was varied. ‘Surgical Attitude to Risk’ included personality as being a significant determinant as to how early surgeons were willing to change their practise. The burden of complications and subsequent possible litigation following complications when a new technique had been used played a role. The final theme was ‘Adopting Evidence in Practise’. These were issues identified with local culture and the actual implementation within a hospital. The issues mentioned were the training individuals received, patient factors and the availability of specific surgical equipment.

Published evidence identifying barriers to the implementation of surgical evidenced based practise have been described elsewhere [17–20]. The scoping review completed as part of this project identified knowledge of the evidence, belief in the evidence, resources and patient factors as possible barriers to implementation of EBM. There is significant overlap with the outcomes of the qualitative analysis which further increases the validity of the work completed. ‘Surgical Attitude to Risk’ is a novel factor identified from the qualitative interviews. Individual surgeons sit on different parts on the adoption curve and so have different threshold for the adoption of new treatments in their clinical practise. There is no broad consensus on the evidence level required for the safest adoption of novel treatments into practise.

In terms of the implications for practice, this study demonstrates that the threshold for which new techniques are introduced appears to vary between surgeons. The STITCH was a multi-centre RCT, however it was the first of its kind. There were surgeons included in this study who changed their practise before the publication of this trial, as a result of the trial and there are those who still remain cynical of the technique and its results. One of the barriers to the uptake of novel techniques is that surgeons will want more evidence than a single study to change their practise. This requires time and significant investment. The role of authoritative bodes was discussed as a possible way to mitigate risk to an individual surgeon, despite EHS guidelines being published years before these interviews took place. Other bodies such as NICE (National Institue for Clinical Excellence) in the UK and ASERNIP-S (Australian Safety and Efficacy Register of New Interventional Procedures) complete reviews and highlight practise that is evidenced based, this is often produced after significant research has been produced and is targeted at the level of operations rather than intra-operative techniques. These different guidelines are mainly produced by surgeons with a significant academic interest and therefore this may mean their perceptions of novel techniques and research data may differ from other surgeons.

The introduction of new techniques is a controversial topic within surgery and the optimum method and timing of implementation of within clinical practise remains unclear. Improving the implementation of high-quality evidenced based work is of upmost importance if we are to reduce research waste and improve outcomes for patients. The IDEAL collaboration is working on the safe introduction of innovation within surgery has led to the creation of a research framework for surgical innovation, however this does not remedy the issue of introduction into routine clinical practise [21]. Rapid dissemination of data prior to publication through online webinars is currently being utilised by surgical research collaboratives and the increasing use of open access journals should also increasingly allow surgeons access to high quality data. There has also been a shift from funding bodies, with a requirement for an implementation plans as part of a submission. The impact of these on the implementation within clinical practice however remains unclear.

This study inevitably had strengths and limitations. This was a well-planned and conducted study with targeted recruitment of surgeons, reflecting over 50% of eligible general surgeons at the research site. There were differences of surgeons interviewed, ensuring that conflicting opinions were identified. There was variation in time of practise, with consultants having been appointed between 2000 and 2016 and surgeons still in training included. There was variation in involvement in academia, reflecting possible differences in literature awareness and accessibility, and belief in the data. The interviewer worked with the interviewees. This may have allowed surgeons to have more open discussions about their practise or alternatively the advocating for the technique from the surgical consultant may have impacted the ability for surgeons to be open. The clinical experience of the research team meant there was a greater understanding of clinical context of the evidence, and this may have led to greater detail within the interviews. A single site was used due to ethical, practical and financial constraints of the project. Surgeons coming from a single institution with the similar experiences, training and culture may have limited the themes identified. Surgical registrars move hospitals as regularly as every 6 months and so were able to provide experience of cultural variation between hospitals.

This study aimed to understand reasons surgeons have and had not changed their practise following the publication of the STITCH trial. This high quality qualitative study identified ‘Trusting the Evidence & Critical Appraisal’, ‘Surgical Attitude to Risk’ and ‘Adopting Evidence in Practise’ as reasons for non-adoption of small bite closure for elective midline laparotomies. These have relevance to other areas of clinical practise and so are areas for potential intervention to increase the uptake of evidenced based surgical practise. Future randomised controlled studies need to include implementation and dissemination plans for the adoption of treatment into routine clinical practise at the point of applying for funding. We know simply publishing a paper will not change practice.

### Electronic supplementary material

Below is the link to the electronic supplementary material.

Supplementary Material 1

## Data Availability

No datasets were generated or analysed during the current study.
