# Supplementary material for: Does randomised evidence alter clinical practise? The react qualitative study
Source: BMC Health Serv Res. 2024 Jul 29;24:862. doi: 10.1186/s12913-024-11305-4 (PMC11287829; doi:10.1186/s12913-024-11305-4)
Supplement: Supplementary file 1 — Supplementary Material 1 [file 12913_2024_11305_MOESM1_ESM.pdf]

# QI Project – REACT

## Semi-Structured Interview Schedule

| Open ended Question | Prompts (use if not covered by initial response) |
|---|---|
| **General demographics and background info** | |
| Gender<br><br>Where and when did you graduate as a doctor?<br><br>What is your area of speciality?<br><br>To what degree are you involved in research yet? | Job plan, attending conferences, publishing, reading journals |
| **Training** | **Prompt** |
| During your surgical training, how were you taught to close a midline laparotomy? | |
| **Closure of Surgical Laparotomy** | **Prompt** |
| Can you tell me how you would go about closing a midline laparotomy wound?<br><br>Is this the way you have always closed a midline wound or has this changed over time?<br><br>Why has this/hasn't this changed?<br><br>What has effected your decision making regarding bite size for midline laparotomy closure? | What size bite do you use? |
| **Culture of Change** | |
| How easy was it/would it be to change your practise?<br><br>To what extent do you feel as though you would need to justify your changes?<br><br>How do you feel about criticisms as a result of complications following a change in practise? | |

| **Evidence Basis** | **Prompt** |
|---|---|
| Have you heard the STITCH trial and what is your knowledge of it?<br><br>Has this affected your decision making regarding closure of midline laparotomy?<br><br>What are your thoughts of the paper? | <br><br><br><br>Positive and Negative |
| **The Future** | **Prompt** |
| What changes to current studies would you have wanted to see in order to change your practise?/ Why has your practised changed following this study compared to others RCTs in other areas?<br><br>What evidence or change in evidence would you need to see in order to change your practise?<br><br>Are you someone who changes their practise a lot and will you continue to do this in the future?<br><br>To what extent do you think surgical training should include aspect of evidenced based medicine?<br><br>Is there anything else you would like to add about anything we have talked about today?<br><br>Do you have any questions about anything we have talked about today? | |
